# Philadelphia Lunatic Asylum, Blockley

**Published:** 1860-01

**Authors:** 


PHILADELPHIA LUNATIC ASYLUM, BLOCKLEY.—Dr. S. W. Butler, one of the editors of the Medical and Surgical Reporter, has been appointed resident physician to the lunatic department of the Philadelphia Hospital, at a salary of $1200 a year. The institution has upwards of 600 inmates.

